# Who benefits from long-term care insurance? Heterogeneous effects among spousal and adult-child caregivers in China

**DOI:** 10.1016/j.ssmph.2026.101976

**Published:** 2026-10-06

**Authors:** Yichen Qiu, Asako Ohinata

**Affiliations:** Department of Economics, School of Business, University of Leicester, 266 London Road, Leicester, LE2 1RQ, United Kingdom

**Keywords:** Long-term care insurance, Caregiver, Health outcome, Policy evaluation

## Abstract

China’s 2016 long-term care insurance (LTCI) pilots represent one of the world’s largest social-care policy experiments. Using the five waves of the China Health and Retirement Longitudinal Study (CHARLS) from 2011 to 2020, we examine how access to LTCI affects the physical and mental health of informal caregivers. We distinguish between spousal caregivers and adult-child caregivers. The latter provide nearly half of all informal care yet have received little attention in the literature. Using a staggered difference-in-differences design, we find that LTCI reduces physical pain for both groups of caregivers, although its effects on their mental health are limited. Our findings highlight the uneven health benefits of social-care reform and underscore the need for policies that provide stronger mental health support for caregivers in ageing societies.

## Highlights

• LTCI significantly reduces physical pain for both adult child and spousal caregivers.
• Caregivers’ physical health gains stem mainly from improved limb and back conditions.
• Spousal caregivers gain most in health with moderately or severely disabled spouses.
• Mental health benefits of LTCI remain limited for both caregiver groups.

## 1 Introduction

Since 2000, China has experienced a sharp increase in its population aged 65 and above, a figure projected to reach nearly 400 million by 2050 (United Nations, Department of Economic and Social Affairs, Population Division, 2024) (see Fig. A1 in the Appendix). Of these, around 66 million people aged 60 and older are expected to require substantial care (World Health Organization, 2015). By 2025, more than 20% of this group may need frequent assistance (Wang, 2024), driving rapid growth in the demand for long-term care (Chen et al., 2021).

Historically, care for older dependent individuals in China has mainly been provided by family members (Feng et al., 2020, Lei et al., 2022). However, demographic shifts and shrinking family sizes have weakened this traditional support system. To address increasing care demands, the government launched a long-term care insurance (LTCI) scheme in select cities from 2012. Funded through existing public medical insurance, LTCI covers those aged 60+ with long-term disabilities, offering daily care and medical support (Lei et al., 2022). The policy primarily provides in-kind services, including home, institutional, and hospital care (Feng et al., 2020, Wang et al., 2023).

In this paper, we study the impacts of Chinese LTCI policy on caregivers’ physical and mental well-being. Although the scheme was intended to support the older individuals in need of care, there is also an important implication for caregivers’ general well-being, since LTCI is likely to reduce the informal care burden. Understanding the impacts on caregivers allows us to evaluate the broader welfare consequences of the policy.

China’s LTCI is expected to improve the physical health of informal caregivers by reducing the intensity of family-provided care and shifting part of the care burden to formal service providers. This may reduce informal caregivers’ physical strain, fatigue, and the cumulative health costs associated with prolonged care provision. International evidence, though limited, generally supports this expectation, showing that long-term care and formal care policies can improve caregivers’ physical well-being by alleviating care burdens (e.g., Fu et al., 2025, Miyawaki et al., 2020).

While this broader literature also suggests that LTCI benefits caregivers’ mental health (Costa-Font and Vilaplana-Prieto, 2022, Juin, 2019, Unger et al., 2021, Verbakel, 2014), the direction and magnitude of the effect in China may be less straightforward. On the one hand, China’s LTCI may improve caregivers’ mental health by reducing out-of-pocket care expenditures and freeing time for rest. On the other hand, these benefits may be attenuated by administrative burdens in accessing benefits or by concerns over the quality of formal care services. More importantly, the Chinese context may differ from that of many other countries because of the continuing strength of filial norms. In China, family members are often expected to take primary responsibility for the care of older relatives. Reliance on formal care may therefore generate feelings of guilt or moral conflict among caregivers. As a result, the Chinese experience may be more complex, and the mental health consequences of LTCI may be weaker or more ambiguous than those observed elsewhere. Evidence from China is also likely to shed light on potential outcomes in other middle-income countries with a similar cultural background or demographic challenges.

Previous Chinese literature highlights how the LTCI policy has significantly influenced the healthcare usage and the outcomes of older Chinese individuals in affected cities. LTCI has contributed to a reduction in the use of healthcare services, including a decrease in outpatient and inpatient visits and associated costs among care recipients (Liu and Hu, 2022, Tang et al., 2022). Furthermore, the policy is reported to have improved the satisfaction with daily living and subjective well-being of the older individuals, contributing to significant improvements in mental health and physical conditions (Chen et al., 2021, Jiang and Yang, 2024, Lei et al., 2022, Tang et al., 2022, Wang, 2024).

Although most studies investigate the impacts on care recipients, a handful of studies show that the benefits extend to spouse caregivers, improving their physical and/or mental health (Jiang and Yang, 2023, Jiang and Yang, 2024, Luo et al., 2024, Yi et al., 2023, Zhang et al., 2025). Their results show that China’s LTCI policy has a positive effect on both the physical and mental health of spousal caregivers. It reduces the number of body pain areas (Yi et al., 2023) and chronic diseases (Jiang & Yang, 2024), alleviates depression (Jiang and Yang, 2023, Luo et al., 2024, Yi et al., 2023, Zhang et al., 2025), and improves self-reported health (Jiang and Yang, 2023, Jiang and Yang, 2024, Luo et al., 2024, Yi et al., 2023) as well as life satisfaction (Luo et al., 2024).

This paper adds to the limited literature on the effects of China’s LTCI policy on caregivers. First, unlike previous studies that focus solely on the health of spouse caregivers, we also examine the impacts on adult children caring for their parents. This is a group that has been completely overlooked in the literature, although approximately half of older dependants in China are cared for by their children and children-in-law (China National Committee on Ageing, 2024). Although spouses often assume the role of primary caregivers and adult children act as secondary caregivers in support of the caregiving parent (Liu et al., 2021), adult children likely face the added burden of balancing care duties with work and family life. Second, in contrast to previous research that only uses a general health index that combines different aspects of physical health, we also analyse specific effects across various body parts, offering clearer insights into the physical toll of caregiving. We also study the behavioural impacts of the policy by evaluating changes in caregivers’ smoking, drinking, and socialisation. Third, we assess the long-term impact of LTCI using panel data from the China Health and Retirement Longitudinal Study (CHARLS), including the latest wave of 2020, extending beyond earlier studies.

Using all available waves (i.e. 2011, 2013, 2015, 2018, 2020) of CHARLS, we estimate the policy impacts on caregiver health using a staggered difference-in-differences approach with a two-way fixed effects model (TWFE). Our estimates indicate that both spouse and adult-child caregivers experience significant improvements in their physical health. However, we observe limited changes in their mental health.

## 2 Background

In 2016, the Chinese government initiated the development of a long-term care insurance system to address the care challenges faced by the ageing population. They selected 15 cities and 2 provinces to pilot its long-term care insurance (LTCI) policy. By September 2020, the number of pilot cities had expanded to 49 (Feng et al., 2020, Li and Otani, 2018). Notably, some cities (e.g. Qingdao and Changchun) had already begun implementing LTCI prior to the national rollout (Luo et al., 2024). A detailed list of cities and the corresponding implementation timelines are provided in Fig. 1.

The LTCI scheme is closely integrated with China’s medical insurance system. It is funded primarily through the Urban Employee Basic Medical Insurance (UEBMI) and the Urban–Rural Resident Basic Medical Insurance (URRBMI) (Pei et al., 2024, Wang et al., 2023). UEBMI covers about 27% of China’s population (National Bureau of Statistics of China, 2025, National Healthcare Security Administration of China, 2025). Although LTCI eligibility generally depends on enrolment in one of these schemes, local policy documents suggest that UEBMI enrollees were more likely to be covered than those enrolled in URRBMI (Feng et al., 2020, Luo et al., 2024). To our knowledge, however, there is no official central government document that clearly and comprehensively summarises this difference across pilot cities. The available evidence instead comes mainly from local policy documents and their syntheses (Chen et al., 2021, Yi et al., 2023).

**Fig. 1:**
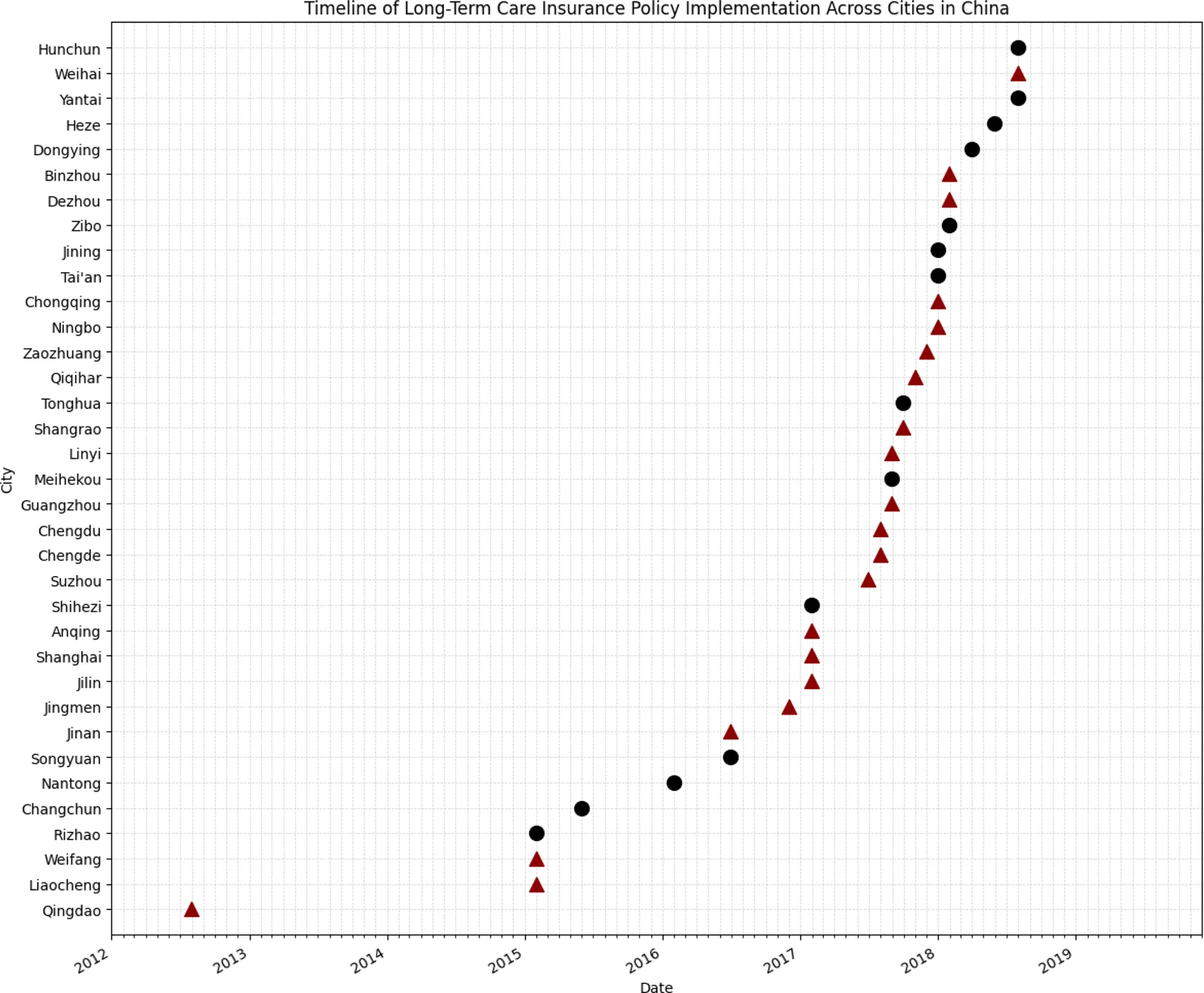
Timeline of long-term care insurance policy implementation across cities in China. *Notes*: The timing of long-term care insurance implementation across cities is determined based on policy documents issued by both central and local government authorities, including agencies such as the People’s Government and the Medical Security Bureau. Relevant documents include *Opinions on Establishing Long-Term Care Insurance* and the *Detailed Rules for the Implementation of the Long-Term Care Insurance Pilot*. Implementation dates may vary depending on the policy documents consulted and the definitions used. To the best of our knowledge, the dates reported here are accurate based on our review of the relevant government documents. Cities indicated by a red triangle are included in the CHARLS dataset, while those marked with a black circle are not.

To qualify for long-term care services, individuals must not only be insured but also meet specific health-related criteria. Eligibility is assessed using the Barthel Index of Activities of Daily Living (ADL) or similar tools developed by each pilot city (Ai et al., 2024, Feng et al., 2020, Lei et al., 2022). The criteria are relatively strict, generally requiring a ‘severe disability’ expected to last at least six months. Only the insured individuals with reduced ability to perform daily activities can apply for an assessment to determine eligibility for LTCI (Ai et al., 2024). Nevertheless, a few pilot cities, such as Shanghai, Suzhou, and Nantong, have broadened their schemes to include those with moderate or even mild disabilities (Luo et al., 2024).

The high cost of formal care in China presents a significant financial barrier, limiting access for many families and exacerbating inequalities in elder care provision. As long-term care is largely financed through out-of-pocket payments, a considerable number of families are unable to afford such services (Liu et al., 2023). For example, home or community-based care typically costs between 50–80 RMB per hour or 2500–17,000 RMB per month (Glinskaya & Feng, 2018). These figures represent approximately 44% to 300% of the average monthly wage (Ai et al., 2024).

China’s LTCI scheme operates on a cost-sharing model. It is primarily funded through the existing medical insurance pool and individual contributions (Wang et al., 2023). In most pilot cities, around 70% of the funding is drawn from medical insurance reserves, while recipients are responsible for the remaining 30% (Feng et al., 2020). This arrangement substantially reduces the financial burden on older adults and their families by offering cost coverage for essential daily care and nursing services costs (Luo et al., 2024, Wang, 2024). By the end of March 2022, a cumulative total of 1.72 million individuals with disabilities had benefited from LTCI, with the average annual payment reaching approximately 16,000 RMB per person (Wang, 2024).

Chinese LTCI provides three main types of benefits: in-kind services, cash allowances, or a combination of both (Luo et al., 2024). All pilot cities offer in-kind services, while a small number, such as Anqing, Binzhou, Shanghai, Shangrao, and Weihai, also provide direct cash subsidies (Lei et al., 2022, Yi et al., 2023). In-kind personal care includes essential daily support, such as feeding or bathing, as well as basic medical services, including nursing care or health consultations.

It is important to remember that the choice of the pilot cities and the implementation timing for the policy may not be random. These cities are selected from different regions with varying levels of economic development and demographic structure (Luo et al., 2024). The choices were also made to ensure equal geographical representation of the eastern, central, and western regions of China (Ai et al., 2024). However, to our knowledge, no official information is available that explains how these cities were selected. If the pilot cities fundamentally differ from the control cities, this would bias our results. We look into this in Section 4.4.

## 3 Data and method

### 3.1 Data

Our dataset combines individual-level data from the China Health and Retirement Longitudinal Study (CHARLS) with city-level characteristics. CHARLS is a nationally representative panel survey of individuals aged 45 and older along with their spouses. It is designed to capture social, economic, and health conditions in China (Zhao et al., 2014). Covering around 20,000 respondents across 28 provinces, the survey is conducted every two to three years through face-to-face computer-assisted interviews. Five waves have been completed (2011, 2013, 2015, 2018, and 2020).

CHARLS primarily surveys two individuals per household, who are typically husband and wife. The timing of LTCI relative to each observation is calculated as the number of months between the survey date and the policy implementation date. To maintain balanced sample size, we group the data into 2-year intervals and restrict the analysis to six years before and after the introduction of the policy.

We identify spouse caregivers using both direct and indirect methods. We only select individuals with spouses in the wave immediately before the policy introduction. We identify the presence of a spouse using the marital status question. Respondents who report being married to a spouse are considered to have a spouse. We exclude individuals who are separated, divorced, widowed, or never married. We also use responses to the Activities of Daily Living (ADL) and Instrumental Activities of Daily Living (IADL) questions. If a respondent reports option 2 (“has difficulty but can still do it”), 3 (“has difficulty and needs help”), or 4 (“cannot complete”) for any of the 11 activities (e.g., eating, bathing, shopping, managing finances) prior to the policy introduction, their spouse is considered a caregiver.

For adult-child caregivers, we include individuals who still have at least one parent alive in the wave immediately before the policy introduction. We used information from the survey question “Is your parent or parent-in-law (mother, father, mother-in-law, or father-in-law) still living?” to determine whether a parent was still alive. This is to ensure that we include individuals who could realistically benefit from LTCI, as deceased care recipients would no longer require or be eligible for such support. We further impose the restriction that the caregiver and care recipient reside nearby in the wave immediately before the policy introduction. We use the survey question “Where do your parents and parents-in-law (mother, father, mother-in-law, or father-in-law) normally live?” for this purpose. Parents are considered to live nearby if they live in the same dwelling, the same village/neighbourhood, or another village/neighbourhood within the same city. The LTCI eligibility depends on the care recipients living in one of the pilot cities, but our sample does not include information on where the parents reside. The last restriction ensures that the individuals in our sample are more likely to look after their parents who benefit from the policy introduction.

We impose separate age restrictions on adult-child and spouse caregivers. Since LTCI eligibility requires care recipients to be 60 years or older, we include adult-child caregivers aged 40 and above and spousal caregivers aged 50 and above. The age 40 cut-off for adult-child caregivers is needed because, although CHARLS officially surveys main respondents aged 45 and older, it does not apply the same restriction to their partners. Having the age 40 cut-off ensures that their parents are likely to be aged 60 or older and thus are potentially eligible for LTCI. We exclude adult-child caregivers older than 80 (8 observations), since they are unlikely to have surviving parents and are themselves more likely to need care. We set the age cut-off for spousal caregivers at 50 to ensure that their frail partners are also more likely to qualify for LTCI. After dropping observations with missing values, the final dataset is an unbalanced panel with 17,897 adult-child caregivers and 13,312 spousal caregivers.

Table 1 presents the definitions of the outcome variables used in the analysis. The dependent variables capture both the physical health and subjective mental well-being of caregivers. Physical health is measured by the number of reported pain areas, representing the total number of body parts in which pain is experienced (including the head, shoulders, arms, wrists, and fingers), with possible scores ranging from 0 to 15. Mental well-being is assessed using the CESD-10 score, which reflects the severity of depressive symptoms; higher scores indicate greater levels of depression (range: 0–30).

In addition to these two main outcomes, we examine several supplementary physical health indicators that identify pain in specific body parts to explore which areas were most affected by the policy. These are binary variables that equal one if the respondent reports pain in a given area (e.g., head, shoulders, or arms). We also examine whether the policy affected caregivers’ behaviours. For this, we analyse smoking, drinking, and social engagement. Our hypothesis is that if the policy improved caregivers’ mental health by reducing caregiving burdens, it may also have reduced smoking and drinking and increased social participation.

**Table 1:** Definition of dependent variables and additional outcomes.

Variables	Description
**Outcome variables**	
Numbers of body areas suffering from pain	Total count of different body parts experiencing pain, summed from head, shoulder, arm, wrist, finger, chest, stomach, back, waist, buttock, leg, knee, ankle, toe, and neck, ranging from 0 to 15.
CESD-10 depression score	Level of depressive symptoms where higher scores indicate greater depressive symptoms, with scores ranging from 0 to 30.
**Additional health and behavioural outcomes**	
Head	Pain in the head. 1 for yes, 0 for no.
Shoulder	Pain in the shoulders. 1 for yes, 0 for no.
Arm	Pain in the arms. 1 for yes, 0 for no.
Wrist	Pain in the wrists. 1 for yes, 0 for no.
Finger	Pain in the fingers. 1 for yes, 0 for no.
Chest	Pain in the chest. 1 for yes, 0 for no.
Stomach	Pain in the stomach. 1 for yes, 0 for no.
Back	Pain in the back. 1 for yes, 0 for no.
Waist	Pain in the waist or lower back. 1 for yes, 0 for no.
Buttock	Pain in the buttocks. 1 for yes, 0 for no.
Leg	Pain in the legs. 1 for yes, 0 for no.
Knee	Pain in the knees. 1 for yes, 0 for no.
Ankle	Pain in the ankles. 1 for yes, 0 for no.
Toe	Pain in the toes. 1 for yes, 0 for no.
Neck	Pain in the neck. 1 for yes, 0 for no.
Smoke	Smokes now. 1 for yes, 0 for no.
Drink	Drinks alcohol in the past year. 1 for yes, 0 for no.
Social engagement	Participated in interactions with friends or community-based organisations during the past month. 1 for yes, 0 for no.

*Notes:* This table includes the list of outcome variables used for our analysis. The variables are taken from the 2011–2020 CHARLS dataset.

Table 2 includes information on the covariates used in our analysis. We incorporate covariates at two levels: individual-level demographic variables and city-year-level variables. Individual-level demographic variables include age, sex, marital status, the number of living children, and the level of education. We also control for indicator variables indicating whether the individual resides in an urban area, has medical insurance, or receives a public pension. Although we would ideally like to control for variables such as the gender composition of caregivers’ siblings or their relationships to the caregivers, our dataset does not include such information.

To account for city-level factors that may influence health outcomes, we include two city-year covariates: annual average PM2.5 concentrations and night-time light intensity. PM2.5, a key air pollutant, is known to harm health and has been linked to an increased risk of depression among the elderly (Aragón et al., 2017, Park et al., 2024). We use satellite-derived PM2.5 data for China from the Atmospheric Composition Analysis Group at Washington University in St. Louis (Van Donkelaar et al., 2019). Night-time light sourced from satellite imagery serves as a proxy for human activity and economic development. We use city-level data from Wu et al. (2021), who produced a harmonised time-series dataset for China by combining DMSP-OLS and SNPP-VIIRS.

**Table 2:** Definition of covariates.

Covariates	Description
**Individual level**	
Age	Respondent’s age.
Female	1 for female, 0 for male.
Married	1 for married, 0 for other (separated, divorced, widowed and never married).
Education level	Seven separate dummy variables for different education levels: no formal education, primary incomplete, elementary school, middle school, high school, vocational school or college, above bachelor.
Urban Area	Current residential area: 0 represents rural area, while 1 represents urban area.
Medical Insurance	Whether the observation is enrolled in any government-operated Medical insurance programme. 1 for yes, 0 for no.
Public Pension	The respondent receive any public pension. 1 for yes, 0 for no.
Number of living children	The number of living children of the respondent.
**City level**	
Pm2.5 mean value	Average level of particulate matter (PM2.5) at the provincial level.
Night light	Logged brightness of visible night light at the city level.

*Notes:* This table includes the list of covariates used for our analysis. The variables are taken from the 2011–2020 CHARLS dataset. The sources of PM2.5 and night light data are https://sites.wustl.edu/acag/datasets/surface-pm2-5/ and https://dataverse.harvard.edu/dataset.xhtml?persistentId=doi:10.7910/DVN/GIYGJU.

**Table 3:** Descriptive analysis of the dependent variables and additional outcomes.

Sample	Adult-child caregivers		Spousal caregivers	
	Mean	SD	Mean	SD
**Dependent variables**				
Numbers of pain areas	1.79	3.09	2.67	3.73
CESD-10 depression score	7.94	6.16	10.06	6.65

Observations	17,897		13,312	

**Additional outcomes**				
**Pain sites**				
Head	0.12	0.32	0.18	0.38
Shoulder	0.13	0.34	0.18	0.38
Arm	0.10	0.30	0.14	0.35
Wrist	0.06	0.25	0.10	0.30
Finger	0.07	0.26	0.11	0.31
Chest	0.05	0.21	0.09	0.28
Stomach	0.08	0.28	0.13	0.33
Back	0.09	0.28	0.14	0.34
Waist	0.19	0.39	0.26	0.44
Buttock	0.04	0.20	0.08	0.27
Leg	0.13	0.33	0.20	0.40
Knee	0.14	0.35	0.22	0.42
Ankle	0.06	0.25	0.11	0.31
Toe	0.04	0.19	0.07	0.26
Neck	0.09	0.29	0.13	0.33

Observations	15,015		10,342	

**Smoke & Drink**				
Smoke	0.40	0.49	0.51	0.50
Drink	0.30	0.46	0.29	0.45

Observations	16,351		12,321	

**Social engagement**				
Social engagement	0.70	0.46	0.68	0.47

Observations	7497		4488	

*Notes*: This table reports descriptive statistics of the outcome variables used in the analysis. The statistics are presented separately for adult-child caregivers and spousal caregivers. Mean values and standard deviations are reported for each variable.

In Table 3, Table 4, we present the mean and standard deviation of the dependent variables and covariates across different sample groups. The average caregiver experiences pain in 2 different areas of the body and has a CESD-10 score of 8.8. A CESD-10 score above 10 is considered indicative of depressive conditions. Spousal caregivers report both a higher average number of pain sites and a higher prevalence of pain in individual body parts than adult-child caregivers. Higher CESD-10 depression score is reported by the Spouse caregivers. When turning to the smoking or drinking behaviours, we observe that spouse caregivers smoke more, but the two groups of caregivers drink similar amounts. These groups seem to have similar social engagement levels.

**Table 4:** Descriptive analysis of covariates.

Covariates	Adult-child caregivers		Spousal caregivers	
	Mean	SD	Mean	SD
Age	56.35	6.72	65.12	8.38
Female	0.52	0.50	0.42	0.49
Married	0.93	0.26	0.96	0.20
*Education level*				
No formal education	0.14	0.35	0.24	0.43
Primary incomplete	0.18	0.38	0.24	0.43
Elementary school	0.22	0.42	0.25	0.43
Middle school	0.28	0.45	0.18	0.38
High school	0.13	0.33	0.05	0.22
Vocational school or College	0.05	0.21	0.03	0.16
Number of living children	2.18	1.04	2.97	1.40
Urban area	0.41	0.49	0.30	0.46
Medical insurance	0.97	0.17	0.97	0.18
Public pension	0.22	0.42	0.50	0.50
Night light	1.48	1.19	1.17	1.21
PM2.5 mean value	43.23	17.90	41.55	17.95

Observations	17,897		13,312	

*Notes:* This table reports descriptive statistics for the covariates used in the analysis. Means and standard deviations are presented separately for adult-child caregivers and spousal caregivers.

**Table 5:** Baseline estimates of static treatment effect.

	Number of pain areas		CESD-10 depression score	
	Adult-child caregivers	Spousal caregivers	Adult-child caregivers	Spousal caregivers
DID_staggered	−0.405***	−0.517**	−0.334*	−0.329
	(0.126)	(0.205)	(0.191)	(0.337)

Observations	17,897	13,312	17,897	13,312
R-squared	0.154	0.164	0.135	0.133
Controls	Yes	Yes	Yes	Yes
Year fixed effects	Yes	Yes	Yes	Yes
City fixed effects	Yes	Yes	Yes	Yes
Cluster level	City	City	City	City

*Notes*: This table reports the baseline estimates of the effects of the LTCI policy on the number of physical pain areas and CESD-10 depression scores. The estimates are calculated using the staggered DID design described in Eq. (1). For adult-child caregivers, the sample is restricted to respondents who: (i) have at least one parent alive in the last pre-policy wave; (ii) have at least one parent living in the same city in the last pre-policy wave; and (iii) are aged between 40 and 80. For spouse caregivers, the sample is restricted to respondents who: (i) have a spouse present in the last pre-policy wave; (ii) whose spouse has functional limitations before the policy, defined as having at least one reported ADL/IADL limitation among the 11 items; and (iii) are aged between 50 and 100. The sample includes 21 LTCI treatment cities and 103 control cities. Standard errors are clustered at the city level and reported in parentheses. Significance levels are indicated by ${}^{*}p<0.1$, ${}^{**}p<0.05$, ${}^{***}p<0.01$.

### 3.2 Empirical method

#### 3.2.1 Baseline model

We estimate the impacts of the LTCI policy on caregivers’ health status by employing a staggered DID design, which we implement using a two-way fixed effects (TWFE) specification:

(1) $$Y_{ict}=\tau D_{ict}+X_{ict}^{\prime }\beta +W_{ct}^{\prime }\delta +\lambda_{t}+\gamma_{c}+\epsilon_{ict}$$

In Eq. (1), $Y_{ict}$ represents the outcome variables (the number of areas suffering from pain and the CESD-10 depression score) for individual i in city c at time t. Our treatment variable, $D_{ict}$, is an indicator that denotes whether the individual i is exposed to the LTCI policy in city c at time t. $X_{ict}^{\prime }$ denotes the individual level covariates: age, gender, marital status, education level, living area, medical insurance, public pension and the number of living children. $W_{ct}^{\prime }$ denotes the city level covariates: PM2.5 mean value and night light. $\lambda_{t}$ is year fixed effects, which capture the time effects. $\gamma_{c}$ represents the city fixed effects, ensuring that they pick up any time-invariant city-specific effects. $\epsilon_{ict}$ is the error term.

#### 3.2.2 Dynamic treatment effect

(2) $$Y_{ict}=X_{ict}^{\prime }\beta +W_{ct}^{\prime }\delta +\lambda_{t}+\gamma_{c}+\left(\underset{t\in \left\{-4,-6\right\}}{\sum }\tau_{t}^{pre}D_{ict}^{pre,t}+\underset{t\in \left\{2,4,6\right\}}{\sum }\tau_{t}^{post}D_{ict}^{post,t}\right)+\epsilon_{ict}$$

In order to interpret τ in Eq. (1) as the causal effect of LTCI on caregiver’s health outcomes, the standard DID parallel trends assumption must hold. Eq. (2) compares the differences in the number of areas suffering from pain and the CESD-10 depression score between the treatment and control groups in each different period related to the benchmark period, which is defined as one period prior to the LTCI policy intervention. t represents different periods before or after the policy, calculated as the difference between the data collection year and the LTCI policy implementation year. $D_{ict}^{pre,t}$ and $D_{ict}^{post,t}$ are indicators for the individual i in city c at time t, denoting respective pre and post policy periods.

$\tau_{t}^{pre}$ measures the treatment “leads”, capturing the effects that occur in the −4 and −6 periods prior to the implementation of the policy. The “leads” serve as the pre-trend event, which helps us test the parallel trends assumption. If a significant difference is observed during the pre-policy period, it indicates that the parallel trends assumption is violated.

When treatment effects are heterogeneous over time, the commonly used Two-Way Fixed Effects (TWFE) model in event studies is likely to produce biased estimates (De Chaisemartin & d’Haultfoeuille, 2020). As noted in Baker et al. (2022), this bias typically manifests itself as a systematic downward bias of the estimates relative to the true average effect of treatment on the treated (ATT), implying that the estimated effects can understate the actual impact of the policy. Moreover, even under the assumptions of homogeneous treatment effects and zero CATTs (Conditional Average Treatment Effects on the Treated) for excluded relative-time periods, event-study specifications that group relative-time periods into bins may still suffer from contamination by CATTs from other periods (Baker et al., 2022). In settings with staggered treatment timing across units, the estimated coefficients for specific leads or lags can be influenced by treatment effects from other periods, and apparent pre-trends may simply reflect underlying treatment effect heterogeneity (Sun & Abraham, 2021). To address these concerns, we also use the estimator proposed by de Chaisemartin et al. (2024), since it is applicable to more general TWFE specifications and is not restricted to any specific treatment assignment structure.

Because LTCI is implemented at the city level, the error terms may be correlated within cities. In all of our specifications, we cluster our standard errors at the city-level.

## 4 Main results

### 4.1 Baseline estimates: Static treatment effect

Table 5 reports the baseline results from the staggered difference-in-differences analysis. We estimate the policy effects separately for spousal caregivers and adult-child caregivers. The results indicate that LTCI leads to significant improvements in physical health for both groups. By contrast, its effects on the CESD-10 depression score are limited, with the estimate for adult-child caregivers being the only one that is marginally significant at the 10% level.

### 4.2 Spatial pain patterns and health behaviours

Although the number of painful body sites used in Table 5 likely proxies for pain severity, this measure does not account for the spatial distribution of pain (Schmidt & Baumeister, 2007). To address this, we examine disaggregated types of physical pain as presented in Panel (a) of Table 6. Both spousal and adult-child caregivers report improvements across almost all body parts. The estimates are particularly strong for the limbs and back, highlighting the policy’s role in alleviating caregivers’ physical pain by reducing the burden of informal care. These results provide further support for the baseline analysis.

We also examine whether the policy affects key health behaviours, such as smoking and drinking, as well as social engagement. Our hypothesis is that if the policy improved caregivers’ mental health by reducing caregiving burdens, it may also have reduced smoking and drinking and increased social participation. At the same time, the results for these outcomes should be interpreted with caution, as they may be jointly determined with depression scores. Social engagement is measured by participation in interactions with friends or community-based organisations during the past month. It is also worth noting that we only use waves 1 to 4 of CHARLS for this part of the analysis, since these questions were not included in Wave 5. In none of the three outcomes, we find significant effects. This is in line with our earlier results that showed limited impacts on the mental health of the caregivers.

**Table 6:** Additional health and behavioural outcomes.

Sample	Adult-child caregivers	Spousal caregivers
**Panel (a) Pain Sites**		
Head	−0.0394** (0.0158)	−0.0403* (0.0223)
Shoulder	−0.0267 (0.0175)	−0.0730*** (0.0219)
Arm	−0.0281* (0.0155)	−0.0551*** (0.0184)
Wrist	−0.0287** (0.0116)	−0.0392*** (0.0118)
Finger	−0.0241** (0.0108)	−0.0290* (0.0153)
Chest	−0.0222** (0.00882)	−0.0289** (0.0130)
Stomach	−0.0230 (0.0154)	−0.0335** (0.0163)
Back	−0.0325** (0.0156)	−0.0365* (0.0198)
Waist	−0.0523* (0.0267)	−0.0275 (0.0224)
Buttock	−0.00887 (0.0133)	−0.00289 (0.0139)
Leg	−0.0435* (0.0236)	−0.0447* (0.0253)
Knee	−0.0412* (0.0215)	−0.0288 (0.0257)
Ankle	−0.0323*** (0.0111)	−0.0276* (0.0158)
Toe	−0.0216*** (0.00616)	−0.0302*** (0.00904)
Neck	−0.0189 (0.0162)	−0.0184 (0.0219)
Observations	15,015	10,342
R-squared	0.166	0.199

**Panel (b) Smoking**		
Smoke	0.00457 (0.00894)	0.0190 (0.0188)
Observations	16,351	12,321
R-squared	0.591	0.529

**Panel (c) Drinking**		
Drink	−0.00602 (0.0132)	−0.0123 (0.0214)
Observations	16,351	12,321
R-squared	0.305	0.230

**Panel (d) Social Engagement**		
Social_engagement	0.0100 (0.0353)	−0.0259 (0.0476)
Observations	7497	4488
R-squared	0.0593	0.0944

*Notes*: This table reports the additional health and behavioural impacts of the Chinese LTCI policy using the 2011–2020 CHARLS data. The sample selection criteria for both adult-child and spousal caregivers are identical to those used in the baseline analysis reported in Table 5. The sample includes 21 LTCI treatment cities and 103 control cities. Standard errors are clustered at the city level and reported in parentheses. Significance levels are indicated by ${}^{*}p<0.1$, ${}^{**}p<0.05$, ${}^{***}p<0.01$.

### 4.3 Heterogeneous treatment effects

We now turn to the heterogeneous analysis by investigating how the policy effects differ across caregivers’ demographic characteristics. Panel (a) of Table 7 shows the same baseline estimates shown in Table 5 for comparison. In line with our baseline results, we observe that caregivers experienced positive effects on their physical health, but limited to no effects on their mental health.

First, we examine heterogeneity by age in panel (b) because the effects of LTCI are likely to vary over the life course. For adult-child caregivers, those below age 60 may be balancing employment and family responsibilities alongside parental care, whereas those aged 60–80 may be more vulnerable to the physical strain of caregiving because of their own declining health. For spousal caregivers, stronger effects may be expected among older individuals, since their partners are also likely to be older and therefore more likely to meet LTCI eligibility criteria. Our physical health results are broadly consistent with this reasoning. Although insignificant, the mental health estimates show a similar pattern.

**Table 7:** Heterogeneous treatment effects: adult-child and spousal caregivers.

	Number of pain areas		CESD-10 Depression	
	Adult-child caregivers	Spousal caregivers	Adult-child caregivers	Spousal caregivers
**Panel (a) Caregiver Type**				
DID_staggered	−0.405***	−0.517**	−0.334*	−0.329
	(0.126)	(0.205)	(0.191)	(0.337)

**Panel (b) Age**				
DID_staggered × age < 60	−0.412***	−0.416	−0.418*	−0.241
	(0.138)	(0.379)	(0.221)	(0.415)
DID_staggered ×60≤ age < 80	−0.396***	−0.572**	−0.215	−0.292
	(0.147)	(0.220)	(0.248)	(0.361)
DID_staggered × age ≥ 80	–	−0.127	–	−1.107
	(.)	(0.637)	(.)	(1.114)

**Panel (c) Gender**				
DID_staggered × male	−0.351***	−0.583***	−0.365	−0.353
	(0.123)	(0.219)	(0.235)	(0.423)
DID_staggered × female	−0.457**	−0.438	−0.304	−0.299
	(0.179)	(0.276)	(0.297)	(0.416)

**Panel (d) Education**				
DID_staggered × Low	−0.402***	−0.511**	−0.335*	−0.316
(high school and below)	(0.127)	(0.209)	(0.192)	(0.336)
DID_staggered × High	−0.492	−0.650	−0.300	−0.631
(vocational and university)	(0.414)	(0.416)	(0.686)	(0.997)

**Panel (e) Number of Living Children**				
DID_staggered × 0 living children	0.720	−0.591	0.179	−2.057
	(1.157)	(1.356)	(2.422)	(1.483)
DID_staggered × 1 living child	−0.403**	−0.748**	−0.228	−0.847
	(0.161)	(0.322)	(0.341)	(0.850)
DID_staggered × 2 or more living children	−0.417***	−0.478**	−0.387	−0.238
	(0.147)	(0.214)	(0.242)	(0.345)

Observations	17,897	13,312	17,897	13,312
Year FE	Yes	Yes	Yes	Yes
City FE	Yes	Yes	Yes	Yes
Cluster level	City	City	City	City

*Notes*: This table presents heterogeneous effects of the LTCI policy on the number of physical pain areas and the CESD-10 depression score. Estimates are reported separately for adult-child caregivers and spousal caregivers. The sample selection criteria for both adult-child and spousal caregivers are identical to those used in the baseline analysis reported in Table 5. The sample includes 21 LTCI treatment cities and 103 control cities. Standard errors are clustered at the city level and reported in parentheses. Significance levels are indicated by ${}^{*}p<0.1$, ${}^{**}p<0.05$, ${}^{***}p<0.01$.

Second, panel (c) shows heterogeneity effects by gender because caregiving roles and burdens may differ systematically between men and women. The descriptive statistics shown in Table 4 highlight that equal proportion of men and women offer care to their parents. Therefore, we do not expect large differences in the policy impact for this caregiver group. In contrast, men are more likely to offer care to their spouses, thus we expect to observe stronger policy impacts among male spouse caregivers. Again, our results support these hypotheses for the impacts on the number of pain areas. A similar pattern is also observed for the impacts on mental health, although the results are again insignificant for the outcome.

Third, we examine heterogeneity by education in panel (d), which may proxy socio-economic resources and access to private coping mechanisms. We define low educated group to include those who have high school education or less. Highly educated group contains those with a vocational or university degree. LTCI may have larger effects among less educated caregivers, who are likely to have limited resources to purchase care privately. The stronger effects among caregivers with a high school education or less are consistent with this interpretation.

Fourth, we consider heterogeneity by number of living children (panel (e)), as this may proxy the amount of family support available to the caregiver. Individuals with no living children may have fewer alternative sources of support. If this is the case, the group could potentially receive the largest benefit from LTCI. However, our results indicate stronger physical health gains among those with more living children. One possible explanation is that these caregivers receive more assistance from their children in navigating the LTCI application process or coordinating care arrangements.

Turning to Table 8, we examine policy effects by the disability level of the care recipient among spousal caregivers. This analysis is only possible for this group, as we do not observe the disability status of older parents in the adult-child caregiver sample. We classify disability as mild for care recipients with at most one ADL/IADL limitation, moderate for those with two to five limitations, and severe for those with six or more limitations. These measures are based on pre-policy information. Therefore, care recipients may be eligible for LTCI later in the post-policy period as their condition deteriorated even if they suffered from moderate disability prior to the policy introduction. The results show that spousal caregivers caring for partners with moderate or severe disabilities experience significant improvements in physical health, consistent with the view that LTCI provides greater relief when care needs are more intensive. By contrast, improvements in mental health, as measured by the depression score, are observed only among caregivers of spouses with moderate disabilities. One possible explanation is that caring for a severely disabled spouse remains psychologically burdensome even after the introduction of formal care support.

**Table 8:** Heterogeneous effects by spouse disability level.

	Spousal Caregivers	
	Number of Pain Areas	CESD-10 Depression Score
DID_staggered × Mild disability	−0.451	0.0715
	(0.328)	(0.489)
DID_staggered × Moderate disability	−0.569**	−0.985**
	(0.236)	(0.465)
DID_staggered × Severe disability	−0.524**	−0.146
	(0.258)	(0.422)

Observations	13,312	13,312
$R^{2}$	0.164	0.133
Year Fixed Effects	Yes	Yes
City Fixed Effects	Yes	Yes
Cluster Level	City	City

*Notes*: This table reports heterogeneous treatment effects by the pre-policy disability level of the spouse care recipient. Mild disability is defined as having at most one ADL/IADL limitation, moderate disability as having two to five limitations, and severe disability as having six or more limitations. These definitions are based on the number of reported ADL/IADL limitations out of 11 items before the policy. The sample selection criteria for both adult-child and spousal caregivers are identical to those used in the baseline analysis reported in Table 5. The sample includes 21 LTCI treatment cities and 103 control cities. Standard errors are clustered at the city level and reported in parentheses. Significance levels are indicated by ${}^{*}p<0.1$, ${}^{**}p<0.05$, ${}^{***}p<0.01$.

**Table 9:** Additional estimates based on LTCI policy details in different pilot cities.

	Number of pain areas		CESD-10 Depression	
	Adult-child caregivers	Spouse caregivers	Adult-child caregivers	Spouse caregivers
**Panel (a) Baseline**				
DID_staggered	−0.405***	−0.517**	−0.334*	−0.329
	(0.126)	(0.205)	(0.191)	(0.337)
Observations	17,897	13,312	17,897	13,312
R-squared	0.154	0.164	0.135	0.133

**Panel (b) LTCI Cities with Severe Disability Eligibility Only**				
DID_staggered	−0.363***	−0.510**	−0.340*	−0.372
	(0.128)	(0.208)	(0.196)	(0.339)
Observations	17,711	13,279	17,711	13,279
R-squared	0.153	0.164	0.133	0.132

**Panel (c) LTCI Benefit Package**				
DID_staggered × In-kind service	−0.443***	−0.475**	−0.417**	−0.449
	(0.133)	(0.227)	(0.197)	(0.297)
DID_staggered × Mixed benefits	−0.246	−0.706*	0.0165	0.218
	(0.267)	(0.367)	(0.429)	(1.251)
Observations	17,897	13,312	17,897	13,312
R-squared	0.154	0.164	0.135	0.133

*Notes*: This table reports additional estimates based on differences in LTCI policy design across pilot cities. Panel (a) presents the baseline results. Panel (b) restricts the treated cities to those that cover only severe disability (i.e. excluding Suzhou, Shanghai, and Nantong, where eligibility also covers individuals with moderate or mild disability). Panel (c) reports heterogeneous effects by LTCI benefit package, distinguishing between in-kind services and mixed benefits (both in-kind services and cash allowances). Cities providing mixed benefits include Anqing, Binzhou, Shanghai, Shangrao, and Weihai, while the remaining pilot cities in our sample provide only in-kind services. The sample selection criteria for both adult-child and spousal caregivers are identical to those used in the baseline analysis reported in Table 5. The sample includes 21 LTCI treatment cities and 103 control cities in Panels (a) and (c). In Panel (b), the treated cities exclude Suzhou, Shanghai, and Nantong. All regressions include city and year fixed effects. Standard errors clustered at the city level are reported in parentheses. Significance levels are indicated by ${}^{*}p<0.1$, ${}^{**}p<0.05$, ${}^{***}p<0.01$.

In Table 9, we also analyse whether we observe differing effects based on the policy details. As discussed in Section 2, most cities only offered coverage for those suffering from severe disabilities whereas individuals residing in three cities (Suzhou, Shanghai, and Nantong) received support even if they only had mild or moderate disabilities. We exclude these three cities to see whether our results are robust to the choice of the pilot cities. The results listed in panel (b) are comparable to those in panel (a) that includes our baseline estimates, reassuring us that our main results are not driven by the choice of pilot cities.

Our final heterogeneity analysis investigates whether policy effects differ by the type of support provided in pilot cities. While most cities offered only in-kind services, a smaller subset (i.e. Anqing, Binzhou, Shanghai, Shangrao, and Weihai) also provided cash benefits alongside in-kind care. The results in panel (c) of Table 9 show stronger effects in cities that offered in-kind support only. This pattern may partly reflect the smaller sample size in cities also offering cash benefits, which is evident in the larger standard errors. It may also indicate that cash payments were used to compensate informal caregivers, rather than to finance formal care, as discussed by Costa-Font et al. (2022). If this is the case, the Chinese LTCI scheme may have generated less substitution from informal to formal care in these cities.

### 4.4 Dynamic effects and parallel trends test

Fig. 2 presents the dynamic effect that also allows us to perform the parallel trends test estimated using Eq. (2). The test was applied separately by the caregiver group and outcome variables. The core assumption of the Difference-in-Differences (DID) method is that, in the absence of treatment, the control group provides a valid counterfactual for the treatment group. However, the government’s selection of pilot cities and the timing of policy implementation may reflect underlying differences in characteristics between the treatment and control groups. If such differences exist, they can violate the assumption of parallel trends and lead to biased estimates.

We examine the pre-policy period to test for the presence of differential trends between the treatment and control groups prior to the implementation of the LTCI policy. The absence of such differences supports the validity of the assumption of parallel trends underlying the DID approach. There were no statistically significant differences in physical pain or depression status between caregivers in the treatment and control groups during the four- and six-year periods prior to the implementation of the LTCI policy. Fig. 2 offers visual images of these estimated results, clearly illustrating the absence of significant pretreatment trends.

**Fig. 2:**
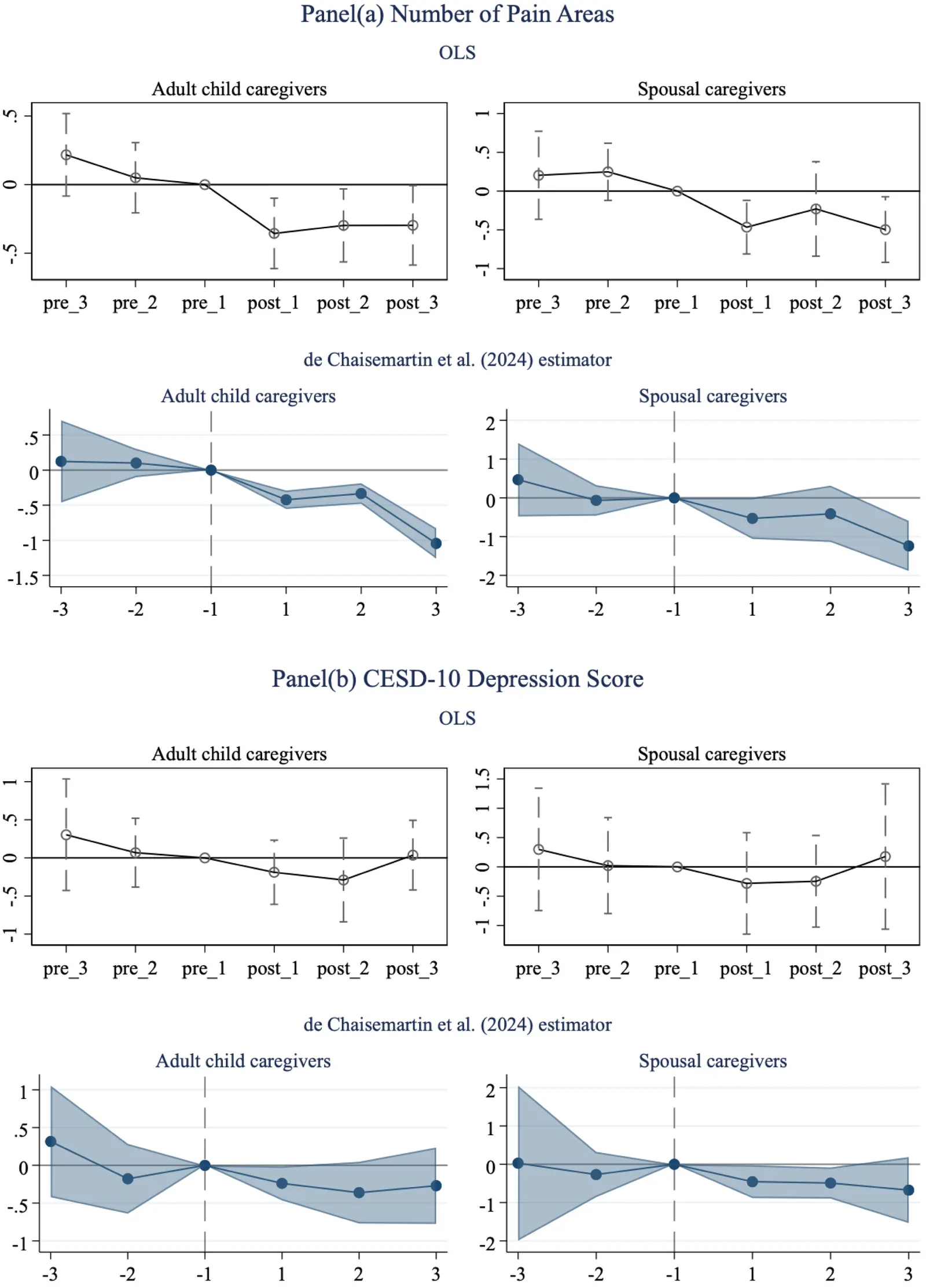
Parallel trend tests and dynamic effects. *Notes*: This figure presents the dynamic impacts of the Chinese LTCI policy. Panel (a) includes the figures for the number of pain sites whereas Panel (b) shows the effects on the CESD-10 depression scores. In each panel, we show the results estimated using the OLS staggered difference-in-differences estimator as well as the estimator proposed by de Chaisemartin et al. (2024). These figures are estimated using the 2011–2020 CHARLS data. 90 percent confidence intervals are presented. Each period on the x-axis represents a 2-year interval. The sample selection criteria for both adult-child and spousal caregivers are identical to those used in the baseline analysis reported in Table 5. The sample includes 21 LTCI treatment cities and 103 control cities. All regressions include city and year fixed effects. Standard errors are clustered at the city level.

In line with the results from Table 5, Table 7, we also find that LTCI significantly reduces the number of self-reported pain areas and this improvement appears to be persistent. However, the impact of LTCI on the depression status of caregivers is minimal. Fig. 2 also applies the estimator by de Chaisemartin et al. (2024), which addresses the potential bias in traditional TWFE models arising from the heterogeneity of the treatment effect over time. Reassuringly, the estimates are broadly in line with those obtained from conventional TWFE regressions.

## 5 Discussion and conclusions

This study provides new evidence on the long-term effects of the 2016 Long-Term Care Insurance (LTCI) policy on the health of Chinese informal caregivers. Unlike the previous studies that exclusively study the impacts on spouse caregivers, our paper also sheds light on the effects on the adult-child caregivers. We use the panel data from five waves of the China Health and Retirement Longitudinal Study (2011–2020) and a staggered difference-in-differences framework.

Our baseline results indicate that both spousal caregivers and adult-child caregivers experience significant reductions in physical pain, while improvements in mental health are limited. Further analysis suggests that the gains in physical well-being are driven by improvements in caregivers’ limb and back conditions, highlighting LTCI’s ability to reduce the physical burden of care by lowering the need to lift or physically assist care recipients. In contrast, we do not observe changes in behaviours such as smoking, drinking, or social engagement. This is consistent with the broader finding that LTCI leads to little improvement in the mental well-being of either caregiver group.

Our estimated policy effects on physical well-being closely resemble those reported by the previous studies. The main difference arises in relation to mental health. While several previous studies report significant improvements in spousal caregivers’ mental health following LTCI implementation, these findings appear to be somewhat sensitive to specification choices. For example, Yi et al. (2023) and Luo et al. (2024) report less significant mental health effects when inference is based on city-level clustering rather than clustering at lower levels. Since LTCI is implemented at the city level, we regard city-level clustering as the more appropriate approach. In addition, some studies, such as Zhang et al. (2025) and Jiang and Yang (2023), include controls for caregivers’ labour market status or the care recipient spouse’s ADL/IADL limitations. While these variables are informative, they may also be jointly determined with caregiver outcomes and therefore affect the magnitude and statistical significance of the estimated coefficients.

It is worth remembering that long-term care needs are inherently heterogeneous, and the effects of LTCI are therefore unlikely to be uniform across caregivers. Both caregivers’ demographic characteristics and the intensity of care recipients’ needs may shape how strongly they benefit from the introduction of formal care support. Our heterogeneity analysis is consistent with this view. We find that the effects of LTCI vary by age, gender, number of living children, and education. For spousal caregivers, stronger effects emerge among older individuals, likely because their partners are more likely to satisfy the age-based eligibility criteria for LTCI and to have greater care needs. Gender differences are limited among adult-child caregivers, where caregiving responsibilities are more evenly distributed, but are more pronounced among spousal caregivers, where men shoulder more care responsibilities.

The heterogeneity results also point to the importance of family resources and socioeconomic background. We find stronger physical health improvements among caregivers with more living children, possibly because a larger family network makes it easier to navigate the LTCI application process and coordinate care arrangements. In addition, LTCI appears to generate larger benefits among less educated caregivers, who are likely to have fewer private resources to substitute for formal care. These findings suggest that the policy may be particularly valuable for caregivers with more limited coping capacity, although access to support within the family may also shape the extent to which these gains can be realised.

For spousal caregivers, we also analyse the effects by the disability level of the care recipient. Physical health improvements are concentrated among those caring for spouses with moderate or severe disabilities, which is consistent with the idea that formal care provides the greatest relief when care needs are more intensive. By contrast, mental health gains are more limited and appear mainly among those caring for spouses with moderate disabilities. One possible interpretation is that, although LTCI can reduce the physical burden of care, the emotional strain associated with caring for a severely disabled spouse may remain substantial even after formal support is introduced. More broadly, these results suggest that simply expanding formal care for frail individuals may not be sufficient to improve caregivers’ mental well-being. If the aim is to support caregivers more comprehensively, additional resources may need to be directed towards those providing informal care, including psychological support, respite services, and other forms of targeted assistance. Taken together, these results reinforce our main conclusion that LTCI can improve caregiver well-being, but that its benefits are unevenly distributed and depend importantly on caregivers’ demographic background and the severity of care needs. This highlights the need for long-term care policy design to account for heterogeneity in caregiving circumstances, particularly if the goal is to support not only physical health but also caregivers’ mental well-being.

**Table A.1:** Baseline results by caregiver type.

	Number of pain areas		CESD-10 depression score	
	Adult-child caregivers	Spousal caregivers	Adult-child caregivers	Spousal caregivers
DID_staggered	−0.405***	−0.517**	−0.334*	−0.329
	(0.126)	(0.205)	(0.191)	(0.337)
Age	0.0239***	−0.0137*	0.0598***	−0.0130
	(0.00572)	(0.00741)	(0.0128)	(0.0136)
Female	0.941***	1.282***	1.977***	2.256***
	(0.0750)	(0.0985)	(0.145)	(0.195)
Married	−0.196*	0.309	−1.788***	−0.643**
	(0.110)	(0.189)	(0.270)	(0.299)
No formal education	0.853***	−0.283	2.720***	1.501
	(0.231)	(0.549)	(0.547)	(0.924)
Primary incomplete	0.904***	−0.290	2.819***	1.622*
	(0.236)	(0.549)	(0.516)	(0.907)
Elementary school	0.727***	−0.495	2.139***	0.744
	(0.220)	(0.536)	(0.487)	(0.919)
Middle school	0.539**	−0.632	1.481***	0.599
	(0.217)	(0.529)	(0.480)	(0.915)
High school	0.600***	−0.500	1.046**	−0.221
	(0.220)	(0.533)	(0.448)	(0.947)
Vocational school or College	0.332	−1.228**	0.380	−2.234**
	(0.210)	(0.545)	(0.527)	(1.037)
Number of living children	0.0521	0.0823**	−0.0386	0.108
	(0.0409)	(0.0353)	(0.0833)	(0.0757)
Urban area	−0.165*	−0.454***	−0.770***	−0.619**
	(0.0925)	(0.118)	(0.219)	(0.299)
Medical insurance	0.0117	−0.165	−0.637**	−0.925**
	(0.133)	(0.170)	(0.281)	(0.370)
Public pension	0.00972	0.120	−0.100	0.157
	(0.0780)	(0.0774)	(0.143)	(0.138)
pm_mean	0.0171**	0.0164	−0.000765	−0.0165
	(0.00799)	(0.00991)	(0.0114)	(0.0135)
log_nightlight	−0.645***	−0.376	0.0134	−0.0653
	(0.210)	(0.322)	(0.296)	(0.430)
_cons	−0.355	2.961***	4.368***	11.06***
	(0.622)	(1.010)	(1.065)	(1.598)

Observations	17,897	13,312	17,897	13,312
R-squared	0.154	0.164	0.135	0.133
Year FE	Yes	Yes	Yes	Yes
City FE	Yes	Yes	Yes	Yes
Cluster level	City	City	City	City

*Notes*: This table includes the baseline estimates shown in Table 5 together with all the covariate estimates. The sample includes 21 LTCI treatment cities and 103 control cities. Standard errors are clustered at the city level and reported in parentheses. Significance levels are indicated by ${}^{*}p<0.1$, ${}^{**}p<0.05$, ${}^{***}p<0.01$.

## CRediT authorship contribution statement

**Yichen Qiu:** Writing – review & editing, Writing – original draft, Visualization, Validation, Software, Methodology, Data curation, Conceptualization. **Asako Ohinata:** Writing – review & editing, Supervision, Methodology, Conceptualization.

## Ethics declaration

Additional informed consent was not required for this study, as it used publicly available secondary data. This study used publicly available secondary data from the China Health and Retirement Longitudinal Study (CHARLS).

This study was performed in compliance with relevant laws, regulatory frameworks and guidelines where the research took place. This study was approved by the University of Leicester. (Approval No. 42512)

## Declaration of competing interest

The authors declare that they have no known competing financial interests or personal relationships that could have appeared to influence the work reported in this paper.

## Figures and Tables

**Fig. A1:**
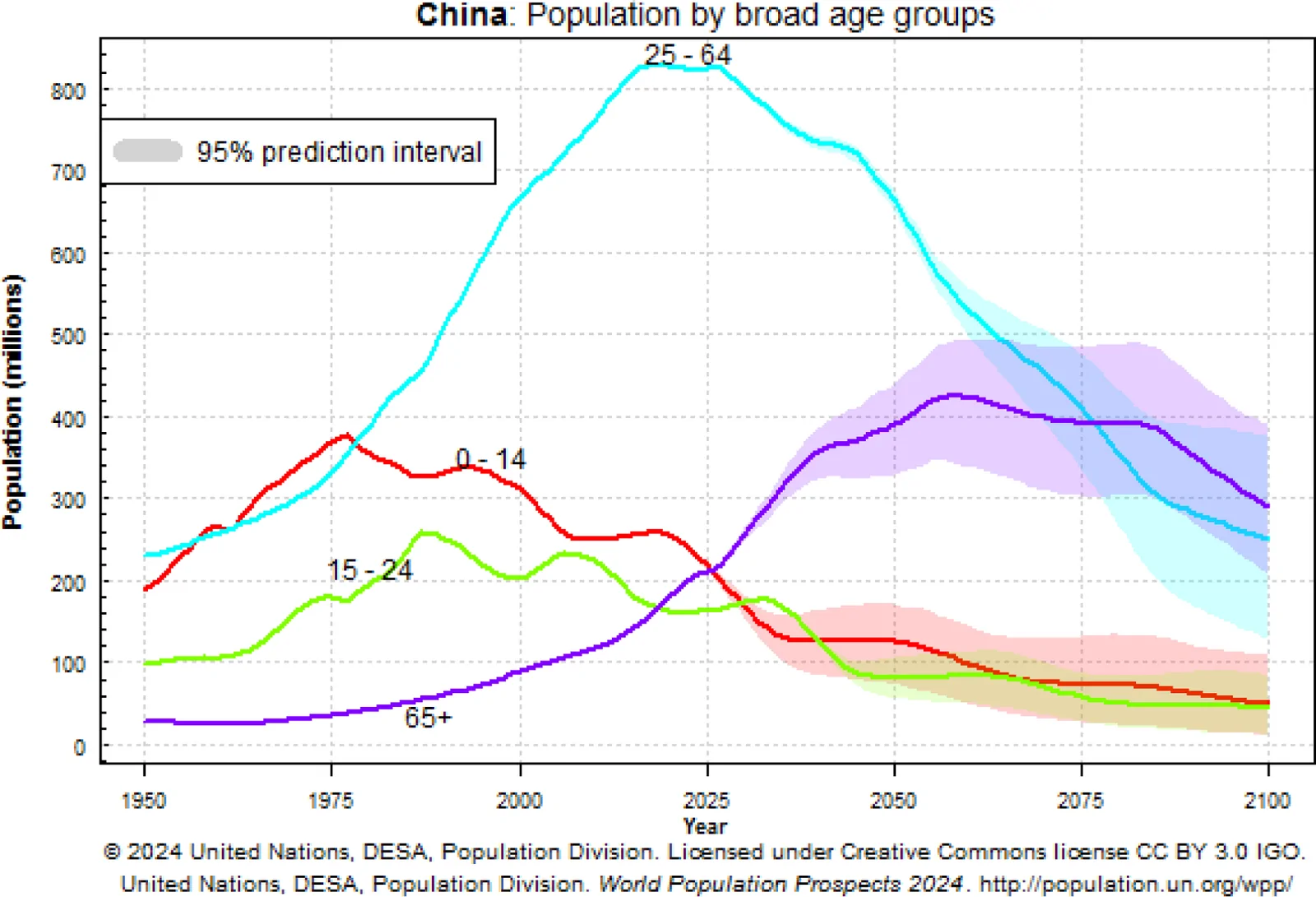
Population by broad age groups in China From 1950–2100 by United Nations, Department of Economic and Social Affairs, Population Division (2024).

## Data Availability

Data will be made available on request.

## Appendix

See Fig. A1 and Table A.1.
